# The Phenol-petrolatum Treatment for Tuberculosis*I have received so many letters referring to my article on “Phenol-petrolatum” as it appeared in the “Red Back,” and as these men continue to show so much interest in the matter, that I beg to submit a short article for publication on this subject.—S. S. W.

**Published:** 1913-09

**Authors:** Stanley Sevier Warren

**Affiliations:** San Angelo, Texas


### TEXAS MEDICAL JOURNAL.

Established July, 1885

F. E. DANIEL, M. D., - - - - Editor, Publisher and Proprietor

Published Monthly.—Subscription, $1.00 a Year.

VOL. XXIX. AUSTIN, SEPTEMBER, 1913. No. 3

The publisher is not responsible for the views of the contributors.

### Original Articles.

For Texas Medical Journal.

### "The Phenol-petrolatum Treatment for Tuberculosis."*

BY STANLEY SEVIER WARREN, M. D., SAN ANGELO, TEXAS.

Any therapeutic agent that is a logical, theoretical, and successful treatment for pulmonary tuberculosis must necessarily be of value, and give equally as good results in glandular and bone tubercular involvements. It is to elicit this proof, and demonstrate its value in the treatment of these conditions, that I submit this brief article to the readers of the "Red Back."

I think that phenol-petrolatum has clearly demonstrated its worth as a therepeutic agent in the treatment of pulmonary tuberculosis, for not only my own results, but the reports that I have received, and am receiving, from physicians throughout the country has clearly demonstrated this.

However, my reports from glandular and bone cases have been meagre, although they have been favorable. It is with the hopes, therefore, that physicians who have such cases under treatment—and who have been unable to get results with other modes of treatment—will administer phenol-petrolatum in these cases, that I am presenting these suggestions.

All of us, of course, realize, and appreciate, that tuberculosis is just as much a constitutional disease as is syphilis; and it would be folly upon the part of any physician to claim for his treatment

*I have received so many letters referring to my article on "Phenol-petrolatum" as it appeared in the "Red Back," and as these men continue to show so much interest in the matter, that I beg to submit a short article for publication on this subject.—S. S. W.

a specific action for any therapeutic agent in a localized, pathological condition, and attempt to excuse its negative value in other local manifestations of the same pathological character.

I have always contended, and still contend, that phenol-petrolatum bears the same relation in its specific action in tuberculosis as do the mercurials in syphilis; and I would as soon think of going before a body of scientific men, questioning the constitutional effects of the mercurials in this disease as to lay any claims for phenol-petrolatum before them with the claim that it was of value only in the treatment of the pulmonary condition. Such a claim would not only be absurdly preposterous, but would at once suggest to any medical man a very great doubt as to its having any value whatever. I make this explanation, and am willing to have the treatment put to this test, because it seems to have been the custom for the originators of any tubercular treatment to claim for their treatment specific action in only one type of tubercular involvement.

It is for this reason, I think, that medical men receive with indifference, and treat with so much skepticism, all new remedies that are offered them in the treatment of tuberculosis. I, myself, am not only willing for phenol-petrolatum to be put to this test, but common sense, and cleancut, logical reasoning tells me that unless phenol-petrolatum will show equally as good results in its curative value in bone and glandular tuberculosis, that the ground is cut from under my feet, and the treatment will receive the same verdict from the medical profession as has every previous treatment.

As for the rationale of the cure, and the therapeutic action of phenol-petrolatum in tuberculosis, I am unable at present to clearly explain. I have submitted this agent to therapeutists of standing and of ability, but up to the present time they have been unable to report clearly and definitely as to the therapeutic action of phenol-petrolatum when administered hypodermatically.

As one man writes me: "It has never been clearly shown, as yet, how or why the mercurials bring about those specific changes in syphilis that check and hold in abatement the ravages of this disease throughout the life of the syphilitic, and yet gives but little, if any, protection to his progeny." He also adds, "That if any specific is ever found for the cure of tuberculosis it is his opinion that their offspring will in nowise be immune from the predisposition to tuberculosis."

While this, of course, has but little bearing upon our present efforts to check and arrest this terrible plague, yet I think these expressions are of much interest to us in showing how the minds of our greatest authorities on questions that concern the advancement of medical science, grasp the minutest detail, and weigh not only results of a treatment that are apparent but take under consideration future development which may or may not prove its value.

It is this faculty of the student, and authority on proposed and existing treatment, that I think, has in a great measure, led to the withdrawal of their endorsement of the tuberculins, and serum treatments for tuberculosis. They have not only recognized the absolute worthlessness of this treatment, but that intuition which is a faculty given to minds of great depth, and thought, has unconsciously led them to dread the evil which it entails. While, I myself, long ago followed the advice of these men, and have discontinued the use of serums and tuberculins, yet I can not help but be impressed with the number of physicians who, when writing me in regard to my treatment, refer to the effects that their patients are enduring from the use of tuberculin.

I quote as an example, from a letter received by me in the morning post, and from a physician: "I have a patient here with tuberculosis who has been out in West Texas, and has had tuberculin given her for several months. She is much worse than when she left here, and it is evident to me, that if some improved method of treating her is not found she is going to die." Out of all the pathetic and pitiful letters that I receive there are none that depress and set me to thinking as do these. Here is a bold indictment, not of our inability and helpnessness as a profession, to cope with this disease, but a direct accusation that a great many of us, at least, are in the face of nature's protest, and against the advice and admonition of recognized authorities administering a proven and discountenanced remedy.

If we can offer nothing of value, it is at least a consolation to know that we can guard nature against this imposition.

In giving out the phenol-petrolatum treatment, I believe that I have probably misled some as to its method of administration, and for that reason I repeat here:

Use the one per cent altogether from the beginning.

Give fifteen minims daily for fifteen days, and fifteen minims every second day for thirty days.

This will constitute a course of thirty doses in forty-five days.

Discontinue the treatment for an interval of from ten to fifteen days.

Then repeat the course in the same way, giving the same dose.

If at any time the expectoration diminishes too rapidly with a rise in temperature, the treatment should be discontinued until the usual amount is being raised and temperature declines.

The injections are given subcutaneously—preferably in the back. You need have no fear of an abscess or any ill effects.

While I would not recommend a stronger incorporation than 1 per cent. yet some physicians have written me that they are using two and five, but until the treatment has become more thoroughly studied by experienced therapeutist, I would advise against this.

Do not look for miraculous results or for a cure when a great amount of lung tissue has been destroyed. Impress it upon your patient that the treatment will have to be given over a period of from four to six months, and persisted in during that time. I explain to my patients that tuberculosis is a slow, wasting disease that nature has been fighting months, and perhaps years, and it will require months to put her back on an equal fighting basis with the disease. Also, that while they will gain strength, the gain in weight will be almost imperceptible.

Caution them above all things, to give up the practice of taking raw-eggs—unless your case is absolutely hopeless. Under these circumstances you may permit the practice.

I wish to repeat that phenol-petrolatum is not the property of any one firm. There is no secret as to what it is, but there is a skill in preparing it, and I recommend that you use only that which is properly made.

I close with the request that you give this treatment a trial in a glandular or bone case.

---

Dinner was a little late. A guest asked the hostess to play something. Seating herself at the piano, the good woman executed a Chopin nocturne with precision. She finished, and there was still an interval of waiting to be bridged. In grim silence she turned to an old gentleman on her right and said: "Would you like a sonata before dinner?" He gave a start of surprise and pleasure "Why, yes, thanks!" he said. "I had a couple on my way here, but I think I could stand another."

---

To encourage the drainage of pus from the pelvis through an abdominal wound it is helpful to have the patient lie face downward at intervals, preferably with the foot of the bed elevated—but not until two or three days after the operation.—*American Journal of Surgery.*

